# WASH Benefits Bangladesh trial: management structure for achieving high coverage in an efficacy trial

**DOI:** 10.1186/s13063-018-2709-1

**Published:** 2018-07-06

**Authors:** Leanne Unicomb, Farzana Begum, Elli Leontsini, Mahbubur Rahman, Sania Ashraf, Abu Mohd Naser, Fosiul A. Nizame, Kaniz Jannat, Faruqe Hussain, Sarker Masud Parvez, Shaila Arman, Moshammot Mobashara, Stephen P. Luby, Peter J. Winch

**Affiliations:** 1 Infectious Disease Division, Environmental Intervention Unit, Enteric and Respiratory Disease Program, International Centre for Diarrhoeal Disease Research, Bangladesh (icddr,b), 68 Shahed Tajuddin Ahmed Sarani, Mohakhali, Dhaka, 1212 Bangladesh; 2 Johns Hopkins Bloomberg School of Public Health, Baltimore, MD USA; 3 Rollins School of Public Health, Emory University, Atlanta, GA USA; 4 Stanford University, Stanford, CA USA

**Keywords:** WASH Benefits trial, Intervention delivery, Behaviour change, Water, Sanitation, Handwashing, Child nutrition, Bangladesh

## Abstract

**Background:**

Water, sanitation, and hygiene (WASH) efficacy trials deliver interventions to the target population under optimal conditions to estimate their effects on outcomes of interest, to inform subsequent selection for inclusion in routine programs. A systematic and intensive approach to intervention delivery is required to achieve the high-level uptake necessary to measure efficacy. We describe the intervention delivery system adopted in the WASH Benefits Bangladesh study, as part of a three-paper series on WASH Benefits Intervention Delivery and Performance.

**Methods:**

Community Health Workers (CHWs) delivered individual and combined WASH and nutrition interventions to 4169 enrolled households in geographically matched clusters. Households were provided with free enabling technologies and supplies, integrated with parallel behaviour-change promotion. Behavioural objectives were drinking treated, safely stored water, safe feces disposal, handwashing with soap at key times, and age-appropriate nutrition behaviours (birth to 24 months). The intervention delivery system built on lessons learned from prior WASH intervention effectiveness, implementation, and formative research studies. We recruited local CHWs, residents of the study villages, through transparent merit-based selection methods, and consultation with community leaders. CHW supervisors received training on direct intervention delivery, then trained their assigned CHWs. CHWs in turn used the technologies in their own homes. Each CHW counseled six to eight intervention households spread across a 0.2–2.2-km radius, with a 1:12 supervisor-to-CHW ratio. CHWs met monthly with supervisor-trainers to exchange experiences and adapt technology and behaviour-change approaches to evolving conditions. Intervention uptake was tracked through fidelity measures, with *a priori* benchmarks necessary for an efficacy study.

**Results:**

Sufficient levels of uptake were attained by the fourth intervention assessment month and sustained throughout the intervention period. Periodic internal CHW monitoring resulted in discontinuation of a small number of low performers.

**Conclusions:**

The intensive intervention delivery system required for an efficacy trial differs in many respects from the system for a routine program. To implement a routine program at scale requires further research on how to optimize the supervisor-to-CHW-to-intervention household ratios, as well as other program costs without compromising program effectiveness.

**Trial registration:**

ClinicalTrials.gov, ID: NCC01590095. Registered on 2 May 2012.

## Background

Water, sanitation, and hygiene (WASH) interventions may include home water treatment, latrine construction, and handwashing promotion, typically promoted by community health workers (CHWs). Due to demonstrated effects of WASH interventions on child health outcomes, such as the effect of handwashing on diarrhoea [1], they have received considerable funding over the years from governments in and donor organizations for low- and middle-income countries [2–5]. However, a considerable research agenda remains regarding (1) the selection of specific WASH technologies and behaviours for inclusion in large-scale, routine programs and (2) how to achieve levels of use and maintenance of technologies that affect sustained adoption of behaviours under routine programmatic conditions sufficient to produce health impact.

Intervention efficacy, effectiveness research and implementation research studies that involve CHWs, particularly to impart behaviour change, differ. There are different uptake goals, CHW-to-population ratios and systems for CHW management and supervision dependent on the size and scope of the study (Table 1). WASH efficacy studies typically examine the effects of specific WASH technologies and behaviors on outcomes of interest in order to inform their subsequent inclusion in routine programs (Table 1). To conduct efficacy studies, CHWs deliver different combinations of WASH interventions to the target population under optimal or ideal conditions (Table 1). In efficacy studies CHWs take great efforts to ensure that the behaviour-enabling technology is present and functional at the household level, and that they are being used (Table 2). This may include free distribution of the technology, and frequent visits by the CHWs to ensure adoption of the behaviours and address difficulties encountered with either the technologies or the behaviours. In contrast, to examine how to achieve high uptake of WASH interventions under near real-world and real-world conditions, researchers build on outcomes from efficacy research and conduct effectiveness research and implementation research (Tables 1 and 2).

**Table 1:** Comparison of uptake goals and implementation procedures for three categories of research on behaviour change interventions delivered by community health workers (CHWs)

Type of research	Validity and causal inferences^1^	Level of control over experimental conditions^1^	Goals for level of uptake^2^	CHW to household ratio	System for CHW management and supervision
Efficacy research	Focus on internal validity: can we draw causal inferences between interventions received and outcomes observed?	Highly controlled, farther from real-world conditions	Technology uptake: optimal Behavioural uptake: optimal	Less than 1/100, not a real-world ratio	Continuous oversight, typically 2–3 times per month. CHWs replaced within 1 month of attrition or critically low performance
Effectiveness research	Focus on external validity: can the results be generalized to programmatic settings with near-real world conditions?	Less controlled, near real-world conditions	Technology uptake: near optimal Behavioural uptake: routine	Ratio based on national Ministry of Health (MOH) policy. Ongoing technical support from NGO staff^3^	Periodic oversight, typical monthly or less. Facilitation of problem resolution by non-governmental organization (NGO)
Implementation research	Focus on external validity: can the results be generalized to programmatic settings with real-world conditions?	Not controlled, real-world conditions	Technology uptake: routine Behavioural uptake: routine	Ratio based on national MOH policy. Limited technical support from NGO staff^4^	Oversight depends on research question Limited external facilitation of problem resolution

^1^Adapted from Curran et al. [10]; ^2^Described in more detail in Table 2; ^3^Typically includes recruitment, training, provision of materials and job aids and on-going supervision; ^4^Typically includes training and provision of behaviour-change communication (BCC) materials and job aids

**Table 2:** Content of behaviour-change interventions delivered by community health workers (CHWs) to achieve different levels of uptake

Desired uptake level	Actions taken by CHWs during intervention delivery to achieve desired uptake level	
	Technology uptake: actions to ensure access to technology	Behaviour uptake: actions to promote behaviour change and technology use
Optimal / ideal	• Technology distributed by CHWs/ project • CHWs provide users with information on use and maintenance of the technology • CHWs conduct frequent home visits to verify that technology is functional and in good working order at all times, and intervene if technology ceases to function	• Behavioural recommendations explained by CHWs during one or more initial home visits • CHWs conduct frequent home visits to verify that people follow behavioural recommendations, and intervene/problem solve if they do not follow the recommendations
Routine	• Technology distributed by CHWs/ project • CHWs provide users with information on use and maintenance of the technology • CHWs do not intervene directly to ensure functionality	• Behavioural recommendations explained by CHWs during one or more initial home visits • CHWs do not intervene directly to ensure that people follow the behavioural recommendations

This article describes the intervention delivery system adopted in the WASH Benefits study in rural Bangladesh, a large-scale efficacy trial of different combinations of WASH and child nutrition behaviour-change interventions delivered by CHWs [6]. The efficacy trial found that nutrition only and nutrition plus WASH interventions improved linear growth and decreased diarrhoea among recipient children [7]. The WASH Benefits interventions were selected from 2 years of pilot studies and the delivery methods aimed at sustained uptake are described in this paper, part of a three-paper series on WASH Benefits Intervention Delivery and Performance [8, 9]; the paper by Rahman et al. [8] describes fidelity measurement methods and monitoring; the paper by Parvez et al. [9] describes methods and findings for intervention uptake. In this paper we describe both the intervention delivery system necessary to achieve the high level of uptake, and the difficulties encountered during intervention delivery and how they were addressed.

## Methods

### Terminology and definitions

We adapt terminology described previously [10], as follows:

- Dissemination: “An effort to communicate tailored information to target audiences with the goal of engagement and information use; dissemination is an inherent part of implementation”
- Intervention: “Health promotion activities being tested or implemented to improve health outcomes.” (adapted from “Clinical Intervention”)
- Intervention delivery (used in place of “Implementation” for the purpose of this manuscript) “An effort specifically designed to get best practice findings and related products into *habitual and sustained use* through appropriate change/uptake/adoption interventions”

For intervention uptake we draw a distinction between actions taken by CHWs to achieve behavioural versus technology uptake and further divide these between optimal and routine uptake to illustrate the differences in CHW-delivered interventions (Table 2).

### WASH Benefits intervention design

**Fig. 1:**
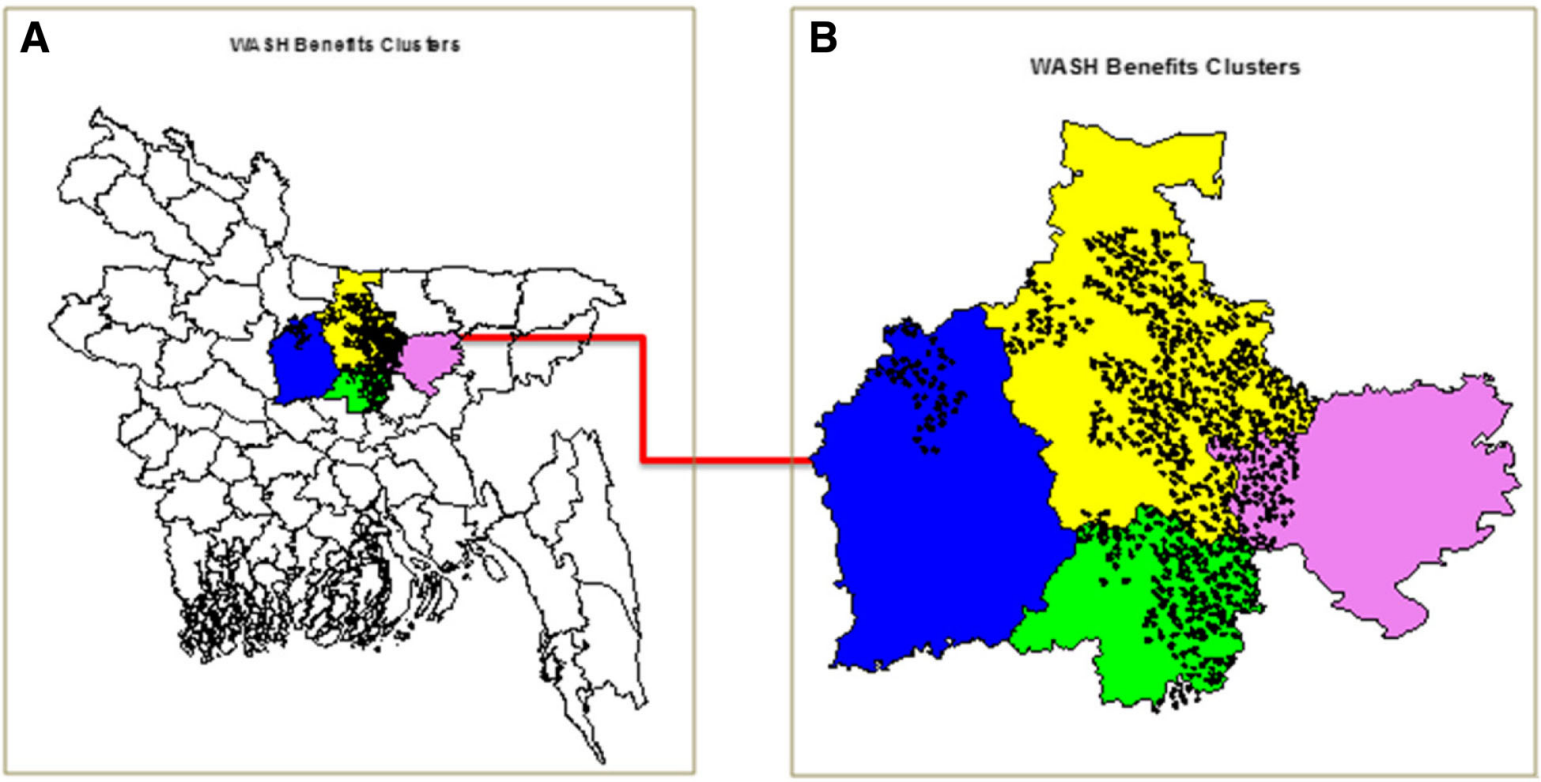
Location of the WASH Benefits Bangladesh Study. **a** A map of Bangladesh showing districts in which the WASH Benefits study was conducted; Tangail (blue), Gazipur (green), Kishoreganj (pink) and Mymensingh (yellow). **b** WASH Benefits study clusters (indicated with black dots) within the four districts

The WASH Benefits Bangladesh cluster randomized controlled trial was conducted in four central Bangladesh districts; Tangail, Gazipur, Kishorgonj, and Mymensingh (Fig. 1). Details of the study design are described elsewhere [6]. In summary, the interventions included free provision of enabling technologies as follows: an insulated drinking-water storage container [11] (Lion Star Plastics, Sri Lanka) for treated stored water; a sani-scoop (a locally developed tool made specifically for the trial, based on previous research [12] was used to remove feces from the household environment; a child’s potty [13] (RFL, Bangladesh) to minimize child open defecation in the household environment; double-pit pour-flush improved latrines [14] for hygienic feces containment; handwashing stations and soapy water storage bottles [15] (RFL Bangladesh). The following supplies were provided for the intervention: Aquatabs™ (Medentech, Wexford, Ireland) for water treatment, laundry detergent sachets for soapy water preparation and Lipid-based Nutrient Supplement (LNS; Nutriset, France). The intervention technologies and products were integrated with parallel behaviour-change promotion, described and displayed in greater detail in the companion paper on monitoring intervention coverage and quality [8]. Behavioural objectives were drinking treated, safely stored water, safe feces disposal, handwashing with soap at key times, and age-appropriate nutrition behaviours (promoted exclusive breastfeeding up to 6 months of age, for children aged 6–24 months complementary feeding and supplementation using LNS). There were six intervention arms comprising 4169 enrolled households in geographically matched clusters: (1) drinking water treatment and safe storage, (2) sanitation, (3) handwashing, (4) combined water + sanitation + handwashing, (5) nutrition, and (6) combined nutrition + water + sanitation + handwashing.

**Fig. 2:**
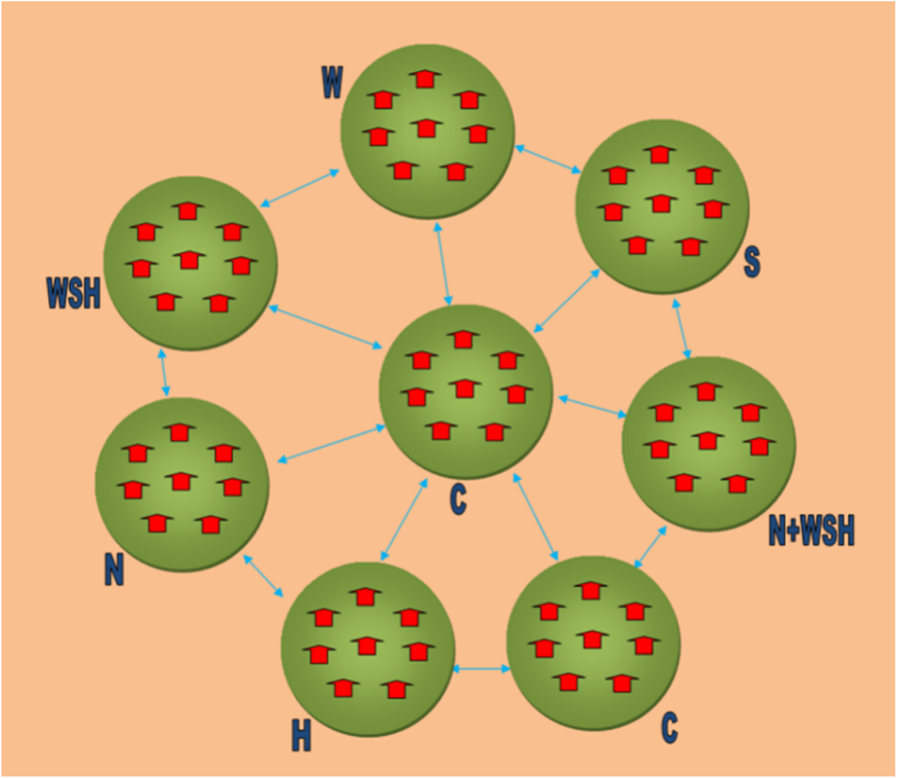
WASH Benefits Bangladesh trial blocks. Each trial block comprised six intervention and two control clusters, each of 6 to 8 households with a pregnant woman (at enrollment) with 1 km buffer between consecutive clusters; *W* water treatment and safe storage, *S* sanitation, *H* handwashing, *N* nutrition, *C* control, *WSH* water treatment + sanitation + handwashing, *N + WSH* nutrition + water treatment + sanitation + handwashing

Each CHW was responsible for one cluster of one intervention arm. Each cluster consisted of six to eight geographically proximate households, identified as having a pregnant mother at enrollment, with an average diameter of 1 km (range 0.2–2.2 km) and being separated from adjacent study clusters by at least 1 km (at least a 15-min walk). The six intervention and two control clusters were grouped into trial blocks (Fig. 2).

### Lessons learned from prior WASH effectiveness, implementation, and formative research studies in Bangladesh

We applied lessons learned from two prior effectiveness and implementation research studies in which several of the WASH Benefits Bangladesh study team members were involved (Table 3).

**Table 3:** Examples of studies of water, sanitation and hygiene interventions conducted in Bangladesh corresponding to the three categories of research on behaviour-change interventions delivered by community health workers (CHWs)

Type of research	Example of this type of research	Scale, setting and duration of study	CHW to household ratio	System for CHW oversight, training and intervention dissemination
Efficacy research	WASH Benefits Bangladesh study^1^	Population; ~ 4169 intervention households; Location: 4 rural sub-districts Duration; 2012–2015 (2-year intervention exposure)	1:8 CHWs; *N* = 540	CHW oversight; Coordinated by study team (Fig. 3) Transparent and rigorous recruitment criteria and methods Identified as employee to maintain morale and prestige in the community Delivery skills formally assessed Accessible supervision, ~ 12 CHWs/supervisor CHWs received study technologies to encourage CHW and recipient self-efficacy Stipend paid using mobile phone network Training: Provided by project staff Refreshers: scheduled and triggered by fidelity measures Included messages on study staff withdrawal and intervention sustainability Dissemination; Based on communication plan and IBM-WASH theoretical model^2^ Evaluated messages were understood by recipients Community meetings during roll out for acceptability, expectation management, conflict reduction Sequenced technology and BCC component delivery for combined interventions
Effectiveness research	Introduction of cholera vaccine in Bangladesh^3^	Population; 240,000 Location: urban slums, Dhaka Duration: 2011–2013 (2-year intervention exposure)	1:290 CHWs; *N* = 55	CHW oversight Single NGO with close project staff involvement, ~ 16 CHWs/supervisor Training Delivered by NGO, developed by project staff Dissemination: NGO-hired CHWs from the local community-delivered interpersonal communications. NGO-delivered hardware
Implementation research	Sanitation, hygiene Education and water supply in Bangladesh Health Impact Study^4^	Population 20 million Location: 58 rural sub-districts Duration: 2007–2012 (4–5-year intervention exposure)	Up to 1:550 CHWs; *N* = 10,000^5^	CHW oversight; multiple NGOs; individual NGOs responsible for hiring and supervision Training: multiple NGOs Dissemination: multiple NGO-hired CHWs from the local community, delivered interpersonal communications

^1^Arnold et al. [6], www.washbenefits.net; ^2^Dreibelbis et al. [21]; ^3^ICVB; the Introduction of Cholera Vaccine in Bangladesh trial [16]; ^4^SHEWA-B; Sanitation, Hygiene Education and Water supply in Bangladesh Health Impact Study [19]; ^5^A total of ~ 10,000 CHWs were engaged in dissemination, but not necessarily the full 5-year intervention period

*NGO* non-governmental organization

The effectiveness study ‘Introduction of Cholera Vaccine in Bangladesh’ (ICVB) [16] included a 2-year handwashing and chlorine water treatment intervention delivered to approximately 240,000 urban low-income household members. The study’s 55 CHWs were recruited and supervised by a single non-governmental organization (NGO). The study team monitored CHW performance, technology, and behavioural uptake (defined in Table 2) and investigated implementation fidelity shortcomings which in turn triggered refresher training sessions for CHWs and/or CHW supervisors in addition to liaison with senior NGO management. However, the water treatment uptake was very low [17]. CHWs did not manage to reach all households as frequently as intended based on workload, faced early confusion about their roles, found enabling technology delivery difficult, and detected problems with quality and provision of technologies and supplies, identified by research field team reports, complicating delivery of the behaviour-change activities.

The Sanitation, Hygiene Education, and Water supply in Bangladesh program (SHEWA-B) included an implementation research component. It evaluated a WASH intervention delivered to 20 million people over 5 years (2007–2012). The program’s 10,000 CHWs delivered behaviour-change promotion, coordinated through 40–60 NGOs that employed, trained, and independently monitored their performance. We detected that CHW visit frequency and health impact were low [2, 18, 19], and that CHWs felt over-burdened, were intermittently paid, and had few training sessions [2, 19].

We therefore aimed to put a system in place that ensured provision of enabling technologies and supplies that were feasible, acceptable, and of a high standard. Technology and supply choices were based on formative research and pilot studies conducted from September 2010 to December 2012. Trials of improved practices compared enabling technology options and included baseline and 3–4-month follow-up surveys to measure uptake, and qualitative studies to assess technology and supply limitations, motivators, and barriers to refine the technologies and the behaviour-change strategy [6, 12, 13, 16, 20] (WASH Benefits Bangladesh Pilot Assessments Report. 2012),). We encouraged behaviour change with a much lower ratio of CHWs-to-households and supportive reminder systems, through an intervention delivery system (Table 3) and described in detail below. We responded to detected implementation fidelity shortcomings [8].

### Intervention delivery system for the WASH Benefits efficacy study

#### Trial oversight

**Fig. 3:**
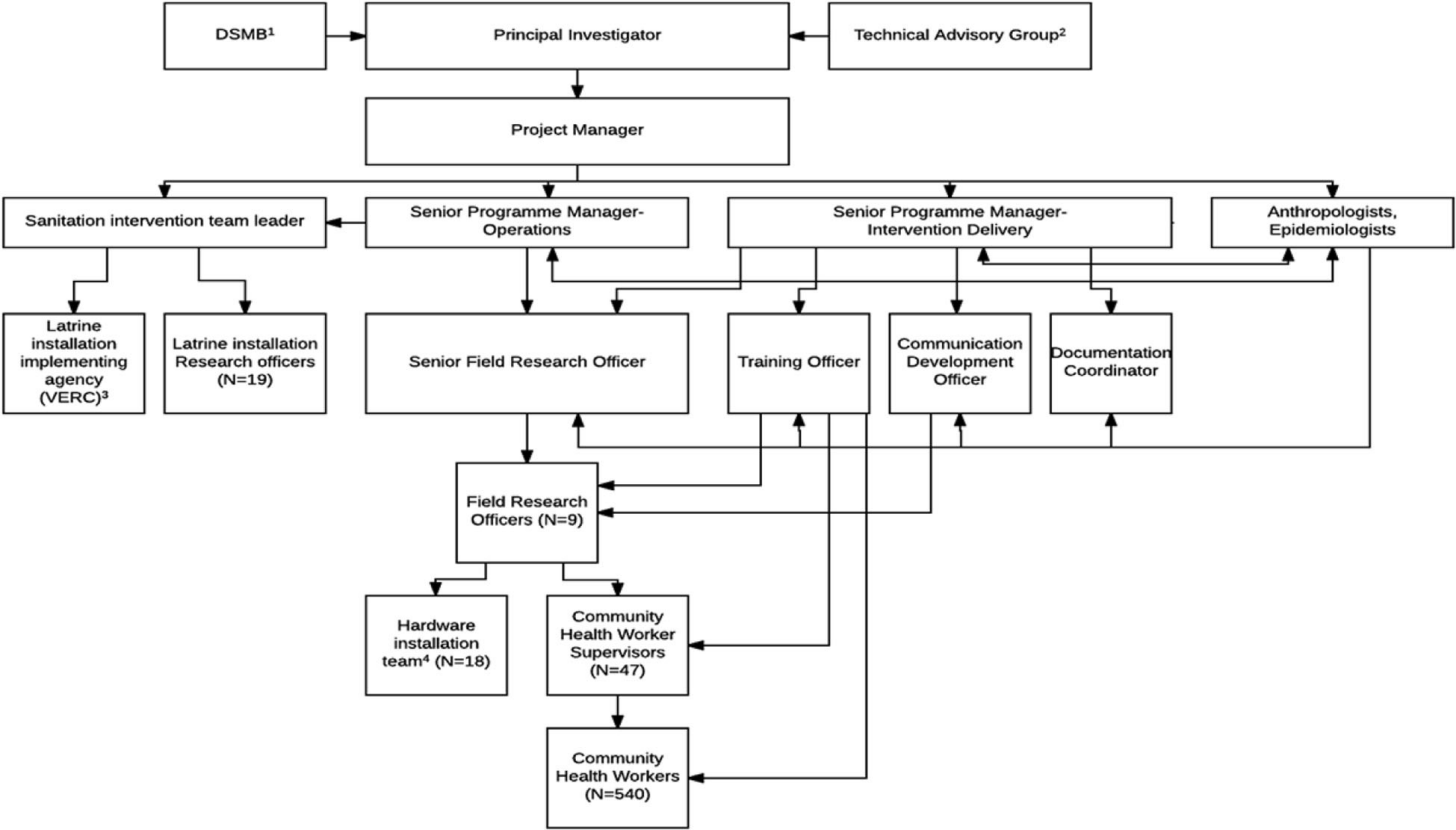
Organizational chart for the Bangladesh WASH Benefits study. ^1^DSMB; Data Safety Management Board; ^2^Technical Advisory Group, comprised members with expertise in anthropology, behavior change, biostatistics, child development, epidemiology, immunology, nutrition, parasitology, and WASH, from Emory University, Innovations for Poverty Action; International Centre for Diarrhoeal Disease Research, Bangladesh; Johns Hopkins Bloomberg School of Public Health; London School of Hygiene and Tropical Medicine; Stanford University; State University of New York; University of California, Davis; University of California, Berkeley; and the Bill & Melinda Gates Foundation; ^3^Village Education Resource Center (www.vercbd.org) ^4^installation of all hardware except latrines

International oversight of the trial was achieved through a central coordinating group at University of California, Berkeley [6], which convened annual meetings from 2009 to 2015 that brought together the Technical Advisory Group (Fig. 3) and the senior scientific and management members of the trial.

Our organizational structure for intervention delivery (Fig. 3) built on lessons learned from the Introduction of Cholera Vaccine in Bangladesh (ICVB) and SHEWA-B intervention delivery structures. The Intervention Delivery Team (comprising a senior program manager-intervention delivery, senior program manager-operations, Sanitation Intervention Team leader, senior field research officer, training officer, field research officers, CHW supervisors, and CHWs; Fig. 3) and the research team (comprising principal investigator, project manager, senior program managers, epidemiologists, anthropologists and collaborators forming the Technical Advisory Group), with the exception of collaborators, were co-located. We managed field intervention delivery through regular team phone calls, field meetings, field reports, and liaison with relevant government and other stakeholders. The Intervention Delivery Team rather than NGO(s) coordinated CHWs to ensure rapid identification of issues with intervention delivery.

#### Community health worker oversight

To recruit local CHWs, the Intervention Delivery Team used transparent selection methods and criteria that included consultation with community leaders, such as teachers and religious leaders, to identified candidate female residents from the study clusters. Candidates completed a written exam and interview. After candidates were offered employment, they were informed of their shared job responsibility, engagement, their pay rate and were provided training. Intervention Delivery Team members described potential benefits of becoming a CHW which included working with a reputable health organization, recognition by the community, gaining skills useful for future employment and they were told that they would receive an employment certificate at the end of their tenure.

CHW supervisors were first trained in direct intervention delivery based on the need to deliver an extensive and intensive intervention training plan. They subsequently trained the CHWs under their supervision (“train the trainer”). Thus, CHW supervisors developed a detailed knowledge of the intervention and its delivery, and a relationship with their team. The field implementation structure ensured that CHW supervisors were highly accessible, by cell phone and through regular visits, maintaining a low supervisor-to-CHW ratio (approximately 1 to 12; Fig. 3). CHW supervisors selected CHWs for regular visit rounds each month, especially for those they thought were under performing or where CHWs had identified uptake issues. Supervisors used a problem solving interactive approach rather than didactic instruction. Moreover, we encouraged informal communications with CHW supervisors by engaging CHWs in a number of social events arranged in the community and the field office to create an environment for interaction. CHWs were trained in technology and supply use, and were given technologies and supplies to use in their own homes, that their recipient household also received.

We developed a Training Plan and Manual for the CHW supervisors to ensure consistency across training sessions. CHW training was conducted using the guiding principles that training was an ongoing process that involved developing a relationship with supervisor-trainers, transferring knowledge through mutual trust and guidance, encouraging participation and developing confidence and ownership of the work. The first round of training included three components: basic training, intervention-specific training and classroom practice/role playing. Basic training included introduction of the project, description of CHW roles and responsibilities, introduction to behaviour-change principles based on the IBM-WASH theoretical framework [9] and interpersonal and counseling communication skills. Intervention-specific training was conducted for 4–9 days; longer duration was required for CHWs delivering combined interventions.

The research team that developed intervention content was also involved in training. Researchers were, therefore, available to respond to questions from CHW supervisor-trainers and CHWs about the rationale for intervention behaviours and technologies. The Intervention Delivery Team included a dedicated trainer (training officer) and a dedicated communication development officer who piloted and produced CHW job aids (Fig. 3). CHWs met with supervisors every month to exchange experiences and learned about the resulting additional or revised behaviour-change activities from the training officer. In addition to these monthly meetings, major refresher training was delivered to all CHW supervisors and CHWs from December 2013 to January 2014, approximately 12–15 months after intervention roll out. CHW work plans were developed with CHW supervisors, during training sessions.

CHWs were provided with institutional ID badges to enhance morale and prestige in the community and had their monthly stipends of approximately US$20, equivalent to 5 days’ full-time salary for a day laborer, delivered through the mobile phone network to ensure timely payments (Table 3).

#### Intervention dissemination

We allocated one CHW to each cluster, usually comprising between six and eight households spread across a 0.2–2.2-km area. Clusters had approximately 1 km between each other to limit intervention spillover.

Dissemination (communicating tailored information to target audiences) was guided by the study behaviour-change strategy, which was based on the IBM-WASH theoretical framework [21] and the prior formative research [6, 12, 13, 16, 20] (WASH Benefits Bangladesh Pilot Assessments Report. 2012)). The study behaviour-change strategy included triggers for refresher training and was used as a method to document intervention revisions. The strategy was modified to include additional promoted behaviours to enhance technology and behavioural uptake (Table 2), recognized during field meetings (manuscript in preparation).

The Intervention Delivery Team conducted community meetings in each intervention cluster, to introduce CHWs, technologies, supplies, and behaviours, and additionally to manage expectations and minimize conflicts identified during the piloting period.

Free provision of technologies and supplies followed a roll-out plan that incorporated actions for hardware repair and replacement, and supply replenishment. For households that received combined interventions, dissemination was sequenced for each intervention component to ensure timely technology and supply delivery and optimal behavioural and technology uptake. During training CHWs were instructed to conduct at least weekly home visits during the first 6 months of the intervention, and at least once monthly visits in the second year.

The dissemination phase began from September 2012 to first 10 trial blocks (Fig. 2), monitored and refined during a 2–3 month period, then rolled out to the remaining trial blocks in nine phases, to accommodate the logistics and the large number of staff members needed to deploy quality interventions (Table 3).

#### Intervention delivery monitoring

We employed several methods to monitor intervention delivery progress. Monthly fidelity measurements were made from 3 months after the intervention was delivered and compared to a *priori* benchmarks. Fidelity indicators were mostly observable proxies for target behaviours, and some household reports, described in detail in the companion paper on monitoring intervention coverage and quality in this three-paper series [8]. In brief, fidelity indicators included the presence of chlorine in stored water, spot checks for the following: sani-scoop accessibility, intact functional household latrine water seal, the presence of water and soap at handwashing locations (near the toilet and the cooking area) and inspection for sachets of LNS consumed. We also obtained data on the following reported indicators: hearing messages on nutrition and/ or LNS, location and method of disposal for child’s last bowel motion. Households were asked to produce the following within 10 s: child potties, sani-scoops, LNS sachets. Structured observations were performed from February to July 2014 (approximately 18 months after dissemination commenced). Observations were conducted among one household randomly selected each from intervention arm (*N* = 6) and from the double sized control (*N* = 2) from each trial block from nine successive implementation phases totalling 324 households. Details on household selection, observation methods, analyses, and direct assessment of behavioural uptake are provided in the companion paper [9].

The senior program manager-intervention delivery, senior program manager-operations and members of the research team that had developed the interventions made periodic field visits, and reviewed monthly reports from intervention delivery staff and CHWs, and from international behaviour-change collaborator visits. Two-weekly calls made from July 2011 to April 2014 among the principal investigator, Bangladesh-based project management staff, and the research team included discussions on intervention delivery troubleshooting and revisions to technology and behaviour-change components; subsequently these were held each month, as issues for discussion lessened.

To measure CHW intervention delivery quality, the Intervention Delivery Team conducted internal CHW performance monitoring and reviewed monthly program reports and staff meeting reports. A donor required process evaluation by an external monitoring agency and this was conducted by the Centre for Research and Management Consulting (SRGB) [10–12].

#### Ethics approval and consent to participate

All households provided written informed consent at enrollment. The protocol was reviewed and approved by human subjects review committees at the International Centre for Diarrhoeal Disease Research, Bangladesh (icddr,b) and at the University of California, Berkeley. The WASH Benefits trial was registered at ClinicalTrials.gov (NCT01590095).

## Results

Implementation monitoring highlighted both successes and shortcomings. Fidelity benchmarks were attained by the fourth intervention assessment month demonstrating moderate to high technology and behavioural uptake, described in the companion paper on monitoring WASH Benefits intervention coverage and quality [8]. This was sustained throughout the program period, described in the companion paper on WASH Benefits intervention uptake [9] for single and combined intervention recipient households. Similar technology and behavioural uptake indicators were detected by the external monitoring agency [22–24]. Periodic internal CHW monitoring resulted in discontinuation of a small number of low performers (*n* = 33). During the intervention period, a total 156 CHWs discontinued service, the most common reason being migration of some type (moved with family, moved abroad, moved after marriage; *n* = 50).

We attempted to address problems as they were detected by the Intervention Delivery Team. The first and most important difficulty was developing a way to manage an intensive training timetable. Considering the complexity of the intervention design and distribution of CHW by intervention type and arm, we initially engaged a professional trainer group to lead the CHW training. They developed the training guideline and communication package and we assigned them to conduct the first batch of training. Despite their expertise in training activities, we observed that the trainer group was not effectively engaging trainees. We found that their highly structured conventional methods likely did not fit with the varied trainee background, particularly for an inexperienced group of selected CHWs. We also understood that CHW motivation would be key and the professional trainers seemed lacking. Thus, we developed an internal training resource group from among our team, thoroughly analyzed the gaps and revised the training methods and materials. Moreover, supervisors were found to be initially minimally engaged with the training and their minimal involvement in training was a lost opportunity for becoming acquainted with intervention detail and developing rapport with the CHWs. Additionally, based on the intervention roll-out timeline, we anticipated that CHW training would take approximately 12–13 months so we used the train the trainer method (described earlier). During plans for training we detected that it was important to further define supervisory roles, hence the development of the Training Manual (described earlier).

Another difficulty was the need to deliver six different interventions, and the potential distance between randomly assigned study clusters. If a CHW had to deliver an intervention in more than one cluster, it would have to be a geographically proximate one; there would have been the potential for CHWs to become confused about message delivery.

Early in the roll-out period, the Intervention Delivery Team detected issues with the pace of hardware installation which could impact the intervention timeline. Latrines were installed for 4533 households and involved numerous construction and quality assurance steps and were thereby delivered independently from the other sanitation technologies and behavioural promotion. Thus, latrine roll out began earlier than other sanitation and other interventions due to an anticipated longer installation period.

To maximize uptake throughout the intervention period, new behaviour-change activities were developed, to address sub-optimal practices detected from structured observations, and to address intervention fatigue reported by CHWs during monthly meetings. Initiatives included further technology use and behaviours that related to the index child age, increasing self-efficacy, increasing roles for men, and decreased emphasis on behaviors that were commonly being practiced. During a 6-month period of political instability, mid-intervention (January to June 2014), we ensured field staff safety through close oversight by the Intervention Delivery Team members at field offices with staff accommodation facilities.

## Discussion

WASH Benefits Bangladesh study intervention delivery built on lessons from prior implementation research [18] and effectiveness research studies [16, 18], to guide development of the intervention delivery system for this efficacy study. As reported in the companion papers in this issue of the journal, the implementation fidelity and uptake data demonstrated that WASH behaviour change met targets considered sufficient for a large-scale efficacy trial [8, 9]. Thus, when interventions were assessed for impact on linear growth and childhood diarrhoea, the WASH Benefits study demonstrated that children from households that received nutrition alone or combined with WASH interventions had significantly greater linear growth. Additionally, children from households that received any of the interventions, with the exception of water treatment, had significantly lower prevalence of diarrhoea. Intervention effects in an efficacy trial cannot be determined when intervention uptake is low.

The high intervention uptake achieved in the WASH Benefits Bangladesh study may be attributed to several factors. Prior formative research and pilot trials provided researchers with some clear choices on attractive, durable enabling technologies and supplies that were provided to enrolled households and would likely encourage behavioural uptake; choices and behaviour-change promotion were underpinned by a theory- and evidence-based behaviour-change strategy. We had conducted community pilots for the ICVB effectiveness study, but behavioural uptake was considerably lower [16]. However, the ICVB pilot period was shortened by an intervention delivery deadline, in line with vaccine delivery, which meant that we were less able to troubleshoot community and household-level problems, particularly with the liquid chlorine-based water treatment intervention. Moreover, in the ICVB intervention, several adjoining unrelated households were expected to share study-provided technologies and supplies [17]. Lower uptake was possibly attributed to additional constraints in urban, low-income environments [13].

CHW-accessible supervision has been highlighted as critical for successful CHW-delivered interventions [25–27]. Particular attention was given to CHW supervision, management, and performance monitoring, based on limitations detected during the SHEWA-B implementation research study, which detected low uptake [2, 19]. SHEWA-B involved more than 10,000 CHWs, each expected to cover 450–550 households [2], who were hired and supervised by 40–60 NGOs during the 5-year intervention period. In an effectiveness research study conducted in India, six different NGOs were contracted to deliver the intervention, and similarly sub-optimal behavioural uptake was found [28]. In the ICVB effectiveness study, a single NGO hired and supervised CHWs, with monthly input from the study research team; however, CHW supervision problems were not totally avoided. In the WASH Benefits Bangladesh efficacy study, the Intervention Delivery Team directly supervised CHWs ensuring frequent interaction, and greater control over intervention delivery.

Training can impact CHW performance [25–27, 29, 30]. The advantages of the training program in this study were that it was developed in collaboration with the research team which had conducted the formative research and pilot studies, and it comprised a train the trainer method for CHW supervisors, longer duration CHW trainings, monthly sessions with the training officer, and refresher sessions driven by adjustments in the behaviour-change strategy. Monthly interactive training meetings that encouraged and acknowledged problem identification likely assisted job satisfaction. In contrast, the SHEWA-B program CHWs received 15 days of initial training in 2007 and 12 total days of refresher training delivered by third-party NGOs between 2009 and 2015 [19].

The supervisor-to-CHW and CHW-to-household ratios in this efficacy study were considerably lower than typical programs and thus facilitated open communication channels. CHW workload has been examined with respect to program delivery quality, and studies have demonstrated that including additional tasks may not compromise quality [31], but heavy workload can impact motivation, performance, and job satisfaction [27]. These indicators were all high among our CHWs, when assessed in a qualitative study (manuscript in preparation).

Non-monetary rewards can contribute to morale and performance [25–27]. For the majority of our CHWs this position was the first paid employment; we used initiatives that we thought would enhance prestige in the community and general job satisfaction while minimizing CHW turnover. We provided ID badges on lanyards, signifying that they were working women and associated with a known and respected health organization. We ensured timely stipend payments through mobile phone networks; frustration over delayed payments was reported by CHWs during the SHEWA-B impact assessment [19].

The intervention delivery system brought together a theory- and evidence-based behaviour-change strategy (Leontsini et al., manuscript in preparation), regular staff interaction at each level of the Intervention Delivery Team, continuous quality improvement principles, and learning from other WASH trials in Bangladesh (Table 3). The co-location of intervention delivery and research staff is relatively uncommon, and likely allowed each group to share and understand the intervention delivery constraints and strengths, ensuring continuous communication and a rapid response to problems identified in the field. An alternative strategy could include assembling research and delivery teams with key program stakeholders in the field to develop and revise a Program Impact Pathway, as described for nutrition interventions [32–34].

An important limitation of our intervention delivery was cost; this was an efficacy trial. Provision of enabling technologies and supplies free of cost to households is not feasible for larger-scale implementation. Opportunities for savings include revising technologies to include simpler hardware, providing technology and product subsidies, or encouraging households to provide some of their own components [35], which may increase feasibility. Intensive behaviour-change activities using frequent one-on-one visits were also expensive and not feasible for larger efficacy trials or implementation programs. However, some aspects of the intervention delivery system, such as communication approaches that focus on developing problem-solving skills rather than didactic transfer of information and lower supervisor-to-CHW ratios could be adopted at a moderate cost.

## Conclusions

Achieving sufficient WASH intervention uptake is attainable; efforts to reduce intervention costs need further exploration. Prior studies have demonstrated that lower intensity is cheaper [36]. However, studies that compare the impact of behavioural and technology uptake at different CHW-visit intensity levels have not been conducted. Regular training that includes office-based interactive sessions with CHWs and their supervisors is costly. Potentially cheaper electronic training (e-training) methods have been employed in high-income countries [37, 38] and will likely appeal to increasingly technology-savvy members of low-income countries where mobile phone and smartphone penetration is high and increasing.

Access to, and interaction with, supervisors has been described previously as impacting on performance [25, 26]. The supervisor-to-CHW and CHW-to-household ratios in WASH Benefits Bangladesh that facilitated open communication channels were considerably lower than typical programs. Exploring effectiveness of higher ratios on CHW performance is needed.

Sustainability of a program beyond program staff presence is an important intervention objective [39], typically addressed in implementation research studies. The extent to which CHW performance impacts uptake beyond the promotion period is an important area for future research.

## Abbreviations

BCC: Behaviour-change communication
CHW: Community health worker
IBM-WASH: Integrated Behavioral Model for Water, Sanitation, and Hygiene
icddr,b: International Centre for Diarrhoeal Disease Research, Bangladesh
ICVB: Introduction of Cholera Vaccine in Bangladesh study
NGO: Non-governmental organization
SHEWA-B: Sanitation, Hygiene Education, and Water supply in Bangladesh program
SRGB: Centre for Research and Management Consulting
WASH: Water, sanitation, and hygiene
